# An Assessment of Time Involved in Pre-test Case Review and Counseling for a Whole Genome Sequencing Clinical Research Program

**DOI:** 10.1007/s10897-014-9697-4

**Published:** 2014-08-01

**Authors:** Janet L. Williams, W. Andrew Faucett, Bethanny Smith-Packard, Monisa Wagner, Marc S. Williams

**Affiliations:** Genomic Medicine Institute, Geisinger Health System, 100 N Academy Ave., Danville, PA 17822 USA

**Keywords:** Whole genome sequencing, Time study, Electronic health record, Genetic counseling, Intellectual disability

## Abstract

Whole genome sequencing (WGS) is being used for evaluation of individuals with undiagnosed disease of suspected genetic origin. Implementing WGS into clinical practice will place an increased burden upon care teams with regard to pre-test patient education and counseling about results. To quantitate the time needed for appropriate pre-test evaluation of participants in WGS testing, we documented the time spent by our clinical research group on various activities related to program preparation, participant screening, and consent prior to WGS. Participants were children or young adults with autism, intellectual or developmental disability, and/or congenital anomalies, who have remained undiagnosed despite previous evaluation, and their biologic parents. Results showed that significant time was spent in securing allocation of clinical research space to counsel participants and families, and in acquisition and review of participant’s medical records. Pre-enrollment chart review identified two individuals with existing diagnoses resulting in savings of $30,000 for the genome sequencing alone, as well as saving hours of personnel time for genome interpretation and communication of WGS results. New WGS programs should plan for costs associated with additional pre-test administrative planning and patient evaluation time that will be required to provide high quality care.

**Electronic supplementary material:**

The online version of this article (doi:10.1007/s10897-014-9697-4) contains supplementary material, which is available to authorized users.

## Introduction

Whole genome and exome sequencing (WGS/WES) are being used for evaluation of individuals with undiagnosed disease of suspected genetic origin (Need et al. 2012; Worthey et al. 2012). Early implementation has involved studies which are conducted as research with return of results. The importance of participant understanding of the broad spectrum of potential results has led to increased time spent in pre-test consenting sessions. A number of publications have described devoting from 2 to 8 h in face-to-face pre-test counseling and informed consent procedures in research and clinical use of WGS/WES (Bick and Dimmock 2011; Tabor et al. 2012). While the articles described pre-test counseling, the time and process used in pre-test intake and evaluation has not been published. As clinical use of WGS/WES becomes increasingly common, it is important to consider the significance of pre-test intake and evaluation and to factor this time and effort into program development. The information gained during pre-test intake and evaluation not only helps to avoid unnecessary genetic testing costs, but also to ensure that the most appropriate patients are being evaluated. This paper presents a quantitative assessment and analysis of the time required for pre-test case preparation, genetic counseling, consenting, and enrollment of families referred to a WGS clinical research program.

## Methods

A whole genome sequencing (WGS) clinical research study at Geisinger Health System, begun in May 2011, enrolled and consented the first family in September of 2012 after receiving IRB approval. The complete program implementation timeline is presented in Table 1.

**Table 1:** Program implementation timeline

WGS program proposed	May 2011
WGS proposal outlined	November 2011
Institutional support obtained	February 2012
IRB review completed	April 2012
Implementation processes developed	August 2012
Initial participant enrollment	September 2012
First sample acquisition for WGS	October 2012
First batched samples to WGS laboratory	November 2012

Participants were referred by Neurodevelopmental Pediatrics, Pediatric Neurology, and/or Medical Genetics to the WGS clinical research program for undiagnosed children. Inclusion criteria consisted of: clinical evaluation and negative genetic testing by a developmental pediatrician, pediatric neurologist and/or medical geneticist within the past 5 years; clinical diagnosis of autism, developmental delay, intellectual disability and/or congenital anomalies; uninformative chromosomal microarray (CMA) testing; and both biological parents available and willing to participate in a trio-based approach to WGS (the proband and both parent genomes are sequenced and parent genomes are used as “filters” to facilitate variant interpretation). An uninformative CMA result was required for participation given the frequency of causal copy number variants (CNVs) in this population, as well as the inability of current next generation sequencing technologies to consistently identify these abnormalities. In addition, most participants had negative results on pertinent diagnostic testing with negative fragile X evaluation, one or more single gene investigation(s), and multiple other diagnostic tests. Exclusion criteria consisted of: non-English speaking parents and families where only one biologic parent was available.

Once referred, the study coordinator (SC) contacted the family to describe the study and screen for eligibility. If the family was eligible and interested, the SC completed the medical history intake (MHI) to summarize the participant’s diagnostic history, requested verbal permission to review the participant’s electronic health record (EHR) to confirm all diagnostic investigations and assessments, noted whether any outside records needed to be requested, and obtained signed release forms, as needed. This information was then reviewed by the medical director who extracted pertinent information into a short anonymous case summary.

This WGS clinical research program was founded on the value of multiple points of review of cases proposed for participation. Therefore case summaries were presented to two program review groups. These groups were put in place to serve as checkpoints to verify participant eligibility, insure that any appropriate testing had already been performed, and make the final decision regarding participant enrollment. The Genome Review Workgroup consisted of study team members and research administrative leadership. Once approved at the Genome Review Workgroup level, the cases were reviewed by the Program Oversight Committee (POC), which consisted of invited physicians from across the health system comprising a variety of pediatric specialties, two community volunteers, and a health plan medical director. At either point of review, recommendations or suggestions could be made to pursue further diagnostic clarification prior to enrollment in the program.

Upon final approval from the POC, the SC notified the family and scheduled a 60 min in-person appointment. Informed consent for the study was obtained by a genetic counselor and the use of a genetic counseling guide (see Supplemental material) facilitated a consistent discussion of WGS with each family. The guide also served as a resource for families to use as a reference after their appointment. Additional follow-up genetic counseling visits were scheduled as needed. The families were also scheduled for an in-person visit with the medical geneticist and for blood draw. Participants who had not had a previous genetics evaluation were scheduled for 60 min and had a dysmorphologic examination. Those who had previously been evaluated by genetics were scheduled for 30 min. If an affected sibling was included, the visit time was extended by an additional 30 or 60 min depending on whether a full dysmorphologic examination was required. Following the appointment with the medical geneticist, blood samples were collected within a Clinical Laboratory Improvement Amendment (CLIA) approved outpatient laboratory and sent to the central institutional laboratory where DNA was extracted, and then samples were sent in batches for WGS to one of three contracted sequencing laboratories.

To track and record the time spent on each step of the study process, the SC maintained Excel files that recorded dates of each contact/interaction with each family, as well as the time spent in program explanation, intake, and medical history collection. The study team also tracked and recorded time spent on committee review of the cases, genetic counseling, consent, physical examination, and sample collection and extraction through the use of thorough meeting minutes, appointment documentation, and discussion with the laboratory technician.

**Fig. 1:**
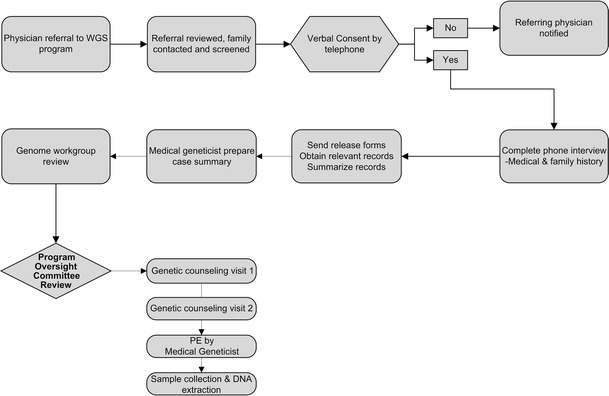
Pre-test WGS clinical research program process diagram. (*PE* physical examination)

The pre-test process is represented schematically in Fig. 1 and provided in greater detail in the Supplemental Materials.

## Results

Neurodevelopmental pediatricians were first to offer this research opportunity to the parents of their undiagnosed patients and to refer families for consideration of WGS. As of May, 2013, 65 families and a total of 75 children (10 sibling pairs) have been referred. The complete WGS clinical research program workflow is shown in Supplemental Figure 1. Of the 53 families contacted to-date, 50 gave verbal consent for access to their child’s Geisinger Health System (GHS) electronic health record (EHR). Two families declined at this point to be considered for inclusion in the WGS program. One family asked to speak with a genetic counselor before proceeding further. After a separate phone discussion with the genetic counselor, the parents declined participation. WGS program participation through 9 months of enrollment has included 29 initial visits for genetic counseling/consenting, 26 subsequent visits completing the genetic counseling/consent process and 26 visits with the medical geneticist. Seventy-five samples for WGS have been collected, DNA extracted and sent to the WGS laboratory.

Throughout this project, it was noted that the time spent on the various program processes fell into two categories—active and passive. “Active” processes were actual time-consuming activities performed by the study team, such as contacting participants, reviewing medical records, examination and genetic counseling. “Passive” processes were steps that resulted in time lags, such as trying to contact participants by phone, waiting for outside records, and scheduling delays.

The average time for each of the program activities is depicted in Table 2.

**Table 2:**
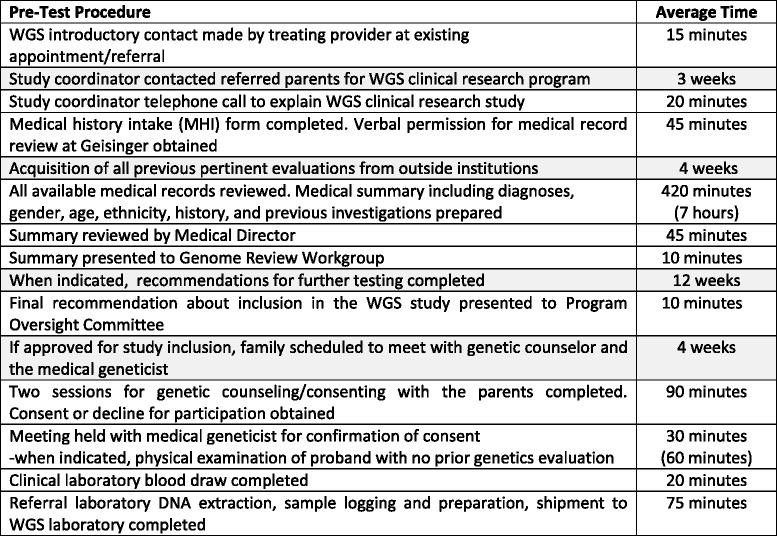
Average time spent on pre-test program processes

“Active” processes are in white, “passive” processes are shaded in gray

### “Active” Processes

The initial phone contact with referred families consisted of approximately 20 min to gauge parental interest, and review the commitment and eligibility requirements. For parents who remained interested, a further 45 min were required by the SC for verbal collection of the participant’s medical history from a parent. If a similarly affected sibling was identified, medical history of the sibling was also noted and potential inclusion in the WGS study was explained. Initial case records review and documentation consumed an average of 8 h. This improved to 7 h as the program progressed, though remained highly dependent upon the extent of the previous diagnostic work-up. Case summary preparation by the medical director averaged 45 min, based on personal report of time tracked from start of case review to completed case summary.

At the beginning of the program, case presentation to the genome workgroup required an average of 15 min of discussion per case. Subsequent POC presentation and review initially required an average 15–20 min of discussion per case. This improved to an average of 7–10 min per case in each group over time, likely due to increased familiarity with the study protocol. POC meeting minutes reflect that one case with specific ethical considerations resulted in lengthy discussion lasting 45 min. This discussion included consideration of the inclusion of a potential participant with mild intellectual disability who was at the oldest age of potential inclusion. The POC acknowledged that the time invested in deliberating issues of appropriate inclusion and informed consent in this case would almost certainly inform similar cases in the future.

Once approved by the POC, the SC scheduled appointments with the genetic counselor and the medical geneticist. Two genetic counselors were involved with this study and coordinated their activities so that each would follow the same family from beginning to end of the study. For all families, genetic counseling visits had an average length of 90 min. The medical geneticist spent either 30 min or 60 min in visit time with the participant child(ren) and parents depending on whether the child had previously been evaluated by a geneticist. Four families included a sibling participant; three of these families did not require a genetics evaluation and were scheduled for 60 min. The other family required examination and thus was scheduled for a 120 min visit time.

The average “active” employee hours expended for pre-test preparation, counseling, examination, consenting, sample collection, and DNA extraction was approximately 13 h per case. An important point to note is that during the chart review process two referred participants were found to have existing, causal diagnoses; the parents were informed and the families were excluded from the WGS program.

### “Passive” Processes

“Passive” processes caused significant program delays. For example, the time required by the SC to connect with newly referred parents by telephone to discuss the WGS clinical research study took, on average, 3 weeks to complete. In a number of cases, signed consents to obtain the results of all previous medical evaluations from outside facilities were obtained. Acquisition of outside medical records took up to 6 weeks and led to significant delays in case preparation time.

Three cases resulted in recommendations from the review groups to pursue other pertinent diagnostic testing and/or review of radiologic imaging studies before proceeding to WGS which delayed enrollment by up to 12 weeks. Once the case received final approval, the SC re-contacted the family to schedule the in-person appointment; the time interval for this contact averaged 4 weeks from approval to first appointment.

Overall, the time from referral to the WGS clinical research program to sample collection averaged 4 months, with a range from 3 months to 9 months.

## Discussion

This study represents the first report of the time required in a WGS clinical research study to contact, inform, consent, and counsel family trios prior to testing. Several previous studies account for the time spent in genetic counseling with families, but there are no reports of preparation time prior to genetic counseling and consent for WGS participation. This study found that the processes involved in preparing cases for the WGS clinical research program could be divided into two categories, “active” or “passive” processes.

Improvements were made decreasing the time spent on “active” processes, such as decreased time spent doing chart review and summarization and time spent by the review committees. This is likely due to increased familiarity with the study protocol and with reviewing the EHR.

However, “passive” processes were less likely to be under the study team’s control and consumed significant amounts of time. These delays included processes such as repeated attempts to reach the participants by telephone during the initial contact and/or during scheduling, and waiting for outside records requests to be fulfilled. This led to delays in the time from referral to sample collection.

Efforts were made to improve the time spent on these “passive” processes. The SC shifted work hours to include more evening hours when possible. This helped to decrease the time needed to connect with families; however, more efficient means to contact families are recommended. While the IRB protocol for this study required initial contact to be completed by telephone, creative use of other modalities such as the patient portal section of the EHR may hold promise.

Significant time delay was experienced in waiting for requested relevant medical records from outside medical institutions that frequently provide sub-specialty services for Geisinger patients. Once the SC identified key contacts at frequently-contacted outside institutions, the forwarding of medical records from those institutions became a more streamlined process with decreased turnaround times noted. However, certain institutions were consistently poor at providing pertinent case-critical laboratory, imaging, or evaluation summaries.

As expected, probands with extensive prior diagnostic investigation required increased time for medical records review and this study illustrates the importance of this in-depth review of medical records. For two participants, the detailed pre-test review process identified a definitive causal diagnosis that had not been reported in the EHR or to the families. One participant had a CNV detected on chromosomal microarray at an outside institution. The result report was included in the records forwarded from the outside institution, however no documentation of communication of the diagnostic result directly to the Geisinger referring physician was found either in the outside records or in the Geisinger Health System (GHS) EHR. For the second diagnosed participant, single gene Sanger sequencing detected a mutation in a causal gene. The ordering physician did not recognize the result as diagnostic and referred the proband for consideration of WGS. This reflects the well-documented issues of genetic test interpretation by providers who do not have specific training in genetics (Baars et al. 2005; Bensend et al. 2013; Giardiello et al. 1997; Plon et al. 2011). This discovery of critical diagnostic information altered involvement in the WGS clinical research program for these families. It is not surprising that communication and/or interpretation failures occur given the sheer number of evaluations that these children undergo at multiple centers. The chart review resulted in savings of $30,000 (the genomes of six individuals, two trios, at a negotiated research cost of $15,000 per trio), not to mention saving hours of personnel time for counseling and consenting prior to WGS, as well as genome interpretation and communication of results after testing.

Finally, various logistical issues also presented unexpected delays to enrolling families in this WGS clinical research program. Examples of logistical issues encountered included the need for clinical space in which to meet and counsel participants and the creation of a clinical research department under which participants could be scheduled according to IRB and compliance requirements. While the specific type and extent of logistical issues will vary by institution, other groups planning to implement a WGS clinical research program will want to plan for such potential clinical research barriers.

### Practice Implications

While the time estimates reported here represent a significant initial investment, thorough pre-test review of cases for WGS eligibility may ultimately result in significant savings of health care dollars and personnel time. In addition, prior work on the interpretation of the WGS results has demonstrated that the upfront investment to precisely define patient phenotypes contributes to decreased time required for the interpretation of results. This approach was used by the winning teams that participated in the CLARITY Challenge, *Children’s Leadership Award for the Reliable Interpretation and Transmission of Your genomic information*, and resulted in honorable mention for the current researchers in this WGS study (Brownstein, CA, 2013, unpublished data). Assessment of the prediction that the pre-test time investment led to decreased time in interpretation will be evaluated once results from WGS become available. Some may argue that the extensive case review was only necessary because of the need to select research candidates, however our team strongly advocates for the careful review of cases, whether in clinical or research applications in order to use an expensive resource wisely and appropriately.

Attention to the pre-test time invested, in addition to the time required for WGS variant interpretation, should continue to be evaluated in order to begin to outline and delineate components of best practices and potential standards of care for WGS.

### Study Limitations

Significant effort by research leadership was expended to facilitate implementation of the WGS clinical research program prior to initial contact of the first family considered for inclusion and was not addressed in this time study.

The time assessments of this study may or may not be generalizable to other institutions, as the present study was conducted within an integrated health care system with full access (upon consent) to participant EHRs which could lead to an underestimate of the time required to complete full medical history review for programs without access to longitudinal EHR data. However, the lessons learned regarding the benefits of thorough chart review and focused genetic counseling may be of use to other groups who plan to begin utilizing clinical WGS.

## Conclusions

Significant time was required to connect with parents to discuss and prepare for participation in this WGS clinical research program. While time improvements were made in “active” study processes, “passive” study processes were often outside the control of the study team. The average pre-test time spent per participant remains at about 4 months, but can range from as short as 3 months to as long as 9 months. Despite improvement, this time investment is beyond that of most clinical activities. However, this study shows that this investment is well-spent, as evidenced by the identification of diagnoses that had not been communicated to families and resulted in significant resource savings and benefit to patient families. The time investment reported in this study is an important formative consideration in the development of programs where implementation of WGS in either clinical or research settings is planned.

## Electronic supplementary material

Below is the link to the electronic supplementary material.

ESM 1 (PDF 447 kb)
