# Supplementary material for: An Assessment of Time Involved in Pre-test Case Review and Counseling for a Whole Genome Sequencing Clinical Research Program
Source: J Genet Couns. 2014 Aug 1;23(4):516–21. doi: 10.1007/s10897-014-9697-4 (PMC4090811; doi:10.1007/s10897-014-9697-4)
Supplement: Supplementary file 1 — (PDF 447 kb) [file 10897_2014_9697_MOESM1_ESM.pdf]

Supplemental Material

An assessment of time involved in pre-test case review and counseling for a Whole Genome Sequencing Clinical Research Program

Janet L. Williams, W. Andrew Faucett, Bethanny Smith-Packard, Monisa Wagner, Marc S. Williams

Genomic Medicine Institute Geisinger Health System Danville, PA

Running head: Time required prior to sequencing in a WGS clinical research program

Corresponding author:

Janet L. Williams

Genomic Medicine Institute

100 N Academy Ave.

Danville, PA 17822

Phone: 570-214-7942

Fax: 570-271-5886

Jlwilliams3@geisinger.edu

Authors' affiliation

JL Williams, WA Faucett, B Smith-Packard, M Wagner, MS Williams

Genomic Medicine Institute

Geisinger Health System

Danville, PA

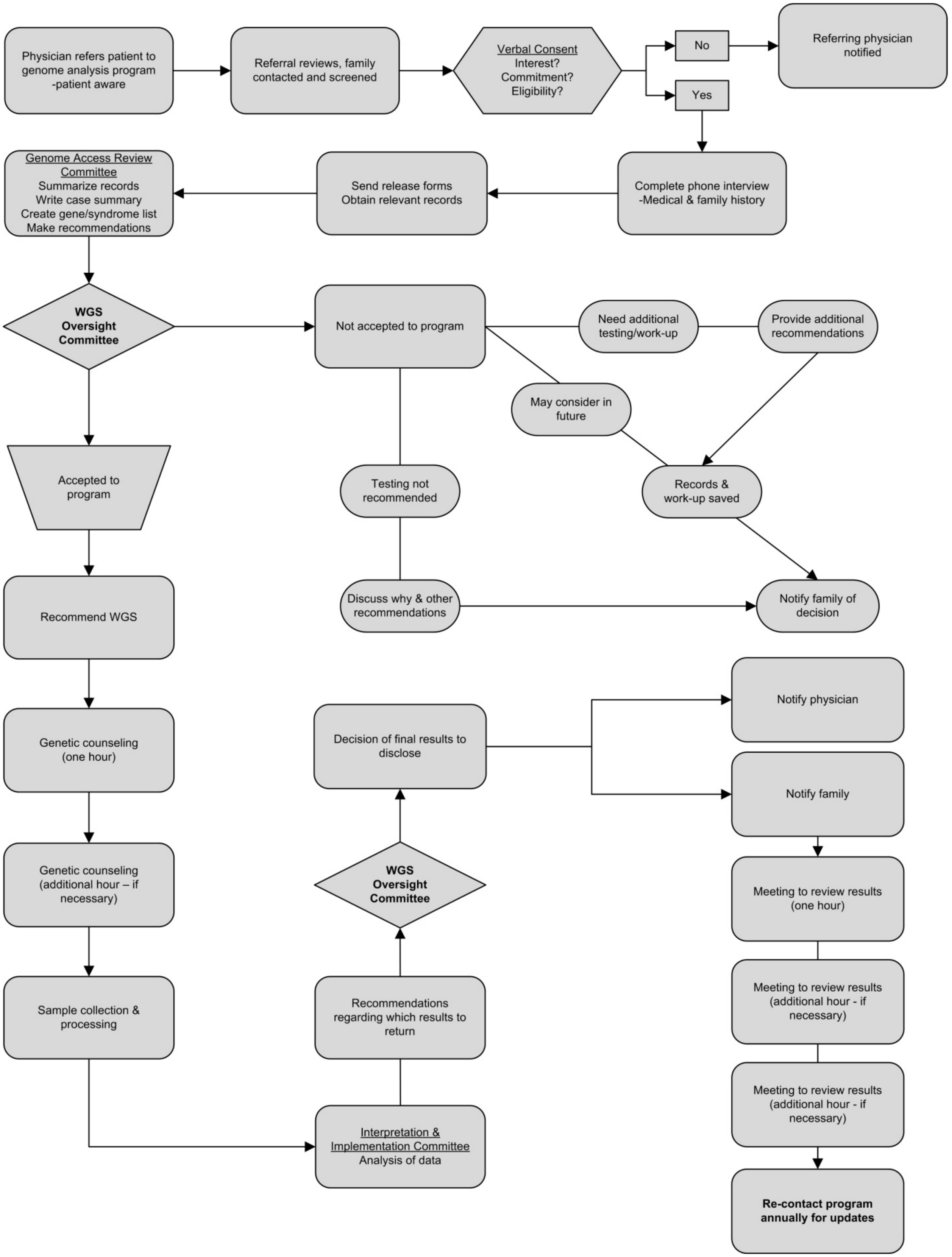

Supplemental Figure 1. Complete WGS research program process diagram.

GEISINGER

Genomic Medicine Institute
Geisinger Health System
100 N. Academy Avenue
Danville, PA 17822-2620

Name:

DOB:

DOS:

Bethanny Smith-Packard, MS, CGC
570-214-9136
Janet L. Williams, MS, CGC
570-214-7942
Monisa Wagner, PA-C
570-214-6197
W. Andy Faucett, MS, CGC
570-214-4862
Marc S. Williams, MD, FACMG

Whole Genome Sequencing Program
Genetic counseling Guide

Background about Whole Genome Sequencing (WGS)

______ Genes contain the genetic material, or DNA, that tells our bodies how to grow and develop. Our genes come in pairs - one copy from our mother, one copy from our father. DNA is the code that makes up how genes are "spelled." Certain changes (mutations) in the DNA code can cause the genes not to work properly and lead to differences in growth and development or to disease. **Whole genome sequencing (WGS) reads through most of the DNA sequence, looking for changes in spelling.**

______ Genetic conditions can be inherited in different ways.
In dominant genetic conditions one non-working copy of a gene causes disease. This mutation can either be passed on from a parent or can be a new change (de novo) in the child. There is a 50% chance to pass on dominant genetic conditions.
In recessive genetic conditions, both copies of a gene must have mutations to cause disease. Therefore, we know that both parents must carry one copy of the mutation. Their other, functional gene protects them from having the condition. Parents who each carry a recessive mutation in the same gene have a 25% chance to have a child with the genetic condition.
In X-linked genetic conditions, only one copy of the gene is needed to cause disease in boys. Since boys only have one X chromosome, they are more likely to show symptoms or have the diagnosis. Since girls have two X chromosomes, a mutation on one X chromosome is protected by the working copy on the other X chromosome. A woman with a mutation on the X chromosome has a 50% chance to pass on this change – if a son inherits the mutation, he would be affected; if a daughter inherits the mutation, she would be a carrier.

______ In addition to the child(ren), both parents are needed for WGS because it allows us to compare the child's DNA sequence to their parents' DNA sequence. **The parents' genomes are used to interpret the child's results** by looking for differences that are new in the child (dominant de novo mutations) or for differences in the same gene that parents carry (recessive and/or x-linked mutations). **While parents' genomes will be sequenced, interpretation of their genomes will not be performed at this time.**

______ **WGS will reveal cases of non-paternity.**

Limitations of WGS

______ While WGS is intended to read through all of the DNA sequence, some regions of the genome can't be read with current approaches. If the cause of your child's problem is in one of these regions it will not be found.

______ WGS will not detect large extra or missing sections of the genome.

______ We know the human body contains about 25,000 genes; we currently understand the function of only about 3,000 genes. In this study, we will focus on the known and understood genes that are likely to be the cause of your child's health concerns.

______ Results will be analyzed by computer programs that use "filters" to narrow down the search for the disease causing gene. During this process, we will not view results that do not meet the filter requirements. Thus, we may not be aware of mutations that have been excluded by this filtering process.

Benefits of WGS

______ Chromosome analysis detects about 5% of genetic causes and microarray detects about 15%. It is estimated that there is an additional 20-40% chance with WGS that we will find a genetic cause for your child's condition.

______ **Finding the genetic cause of your child's health concerns may or may not lead to a change in your child's care or treatment.**

______ Participating in this program may help other families with undiagnosed conditions and benefit general knowledge about genetic disease.

______ WGS results will need to be reanalyzed in the future as new information is learned about the human genome. This will also address some of the limitations noted above.

______ **There may not be a direct benefit to your child.**

Risks of WGS

______ You will be required to provide a blood sample. Anytime you have blood drawn, there may be very minor risks, including brief pain, slight bruising, dizziness or fainting, and (very rarely) infection where the needle is inserted.

______ The testing process may reveal unexpected or upsetting results not related to your child's diagnosis, for example, a mutation in one of your genes that could predict future health problems for you or indicate an increased risk in a future pregnancy for you or other family members. This risk is low since the analysis process will focus on finding genetic changes that explain your child's condition. **The program intends to report all results that are believed to be clinically relevant (see below).**

______ If results are found that could impact your family members, we will talk with you about how to communicate this information to relatives.

______ **There is a chance that a diagnosis will not be found through this program.** While WGS is expected to provide many answers, there are still limitations in the technology and in how we are able to interpret the data it provides.

______ Results from WGS may lead to more investigations, testing, procedures, or increased/additional follow-up for your child or family.

______ Results of WGS may reveal that parents are related.

______ While there is a risk for loss of confidentiality, none of your Protected Health Information (PHI) will be shared outside Geisinger.

Type of Results Possible

______ While the whole genome will be sequenced, this program will focus on genes related to your child's health concerns and his/her diagnosis. Types of results may include:

- No mutations found to explain your child's diagnosis
- Mutation(s) found that explain your child's diagnosis
- Mutation(s) of uncertain significance – unable to be interpreted at this time
- Secondary results, unrelated to your child's current health concerns, but considered important for his/her care

______ If no explanation for your child's health problem is found, there may be the opportunity to participate in other research projects.

Type of Results Shared

______ We will share results with you that focus on the reason for WGS (your child's health concerns and diagnoses).

______ In the future we will share other results that may lead to a change in your child or your family's medical treatment, management or reproductive risk. Examples of these types of results include childhood onset medical conditions, adult onset medical conditions, results that affect the use of medications (pharmacogenetic results), and carrier status for conditions where genetic screening is recommended by the American College of Obstetrics and Gynecology (ACOG).

______ In the future, parents may choose to have their individual genome results analyzed separately for an additional charge.

______ In the future, families may be offered the option to have genetic carrier status analyzed for conditions not currently recommended for screening by ACOG for an additional charge.

GEISINGER

Type of Results Not Shared

______ This program will not report:

- Adult onset conditions with no known treatment
- Variants in a gene not known to cause disease
- Results of uncertain significance

Testing Process

______ At this time, the WGS testing process takes about 4 months to perform the DNA sequencing. After DNA is sequenced, analysis and interpretation will take about 4 months.

______ All WGS results that will be shared with you will have been confirmed in a CLIA-certified (clinical) laboratory and will have been reviewed by the program oversight committee before being shared with you.

______ There are no charges for participating in the Whole Genome Sequencing Program. Your insurance will not be billed. If you receive a bill, please call us immediately.

Discussion of Results

______ Results will be discussed in person. When results are being finalized, we will contact you to set up a genetic counseling visit to discuss results of WGS. You may have one or more appointments depending on how many results are found and how many questions you have.

Follow-up

______ Your WGS results will be available for reanalysis in the future as we continue to learn about the genome. The frequency has not been determined at this time but will depend on your results and what new information we learn in the future. We may also decide to reanalyze the results if you report a new diagnosis in the family that may aid our interpretation of the results. **For this reason, you should keep in contact with the WGS program on a yearly basis.**

Storage of Data

______ **After testing is completed, your DNA sample will be retained, without any identifying information, for research purposes. Your de-identified results may be submitted to a research database where only approved investigators may access this information, unless otherwise indicated below. Choosing not to participate in medical research will not affect your results.**

☐ **I do NOT consent to the use of my DNA sample and results for research purposes.**

Date of next appointment: ____________________ Date of blood draw: __________________________

____ ____

Mother's signature Father's signature

____ ____________________ _______________

Genetic Counselor's signature Date Length of Visit
